# The genome‐wide expression effects of escitalopram and its relationship to neurogenesis, hippocampal volume, and antidepressant response

**DOI:** 10.1002/ajmg.b.32532

**Published:** 2017-04-10

**Authors:** Timothy R. Powell, Tytus Murphy, Simone de Jong, Sang Hyuck Lee, Katherine E. Tansey, Karen Hodgson, Rudolf Uher, Jack Price, Sandrine Thuret, Gerome Breen

**Affiliations:** ^1^ King's College London, Social, Genetic and Developmental PsychiatryInstitute of Psychiatry, Psychology and Neuroscience (IoPPN)LondonUnited Kingdom; ^2^ National Institute for Health Research Biomedical Research Centre for Mental Health, Institute of PsychiatryPsychology and Neuroscience at the Maudsley Hospital and King's College LondonLondonUnited Kingdom; ^3^ King's College London, Department of Basic and Clinical NeuroscienceInstitute of Psychiatry, Psychology and Neuroscience (IoPPN)LondonUnited Kingdom; ^4^ College of Biomedical and Life SciencesCardiff UniversityCardiffUnited Kingdom; ^5^ Department of PsychiatryDalhousie UniversityHalifaxCanada

**Keywords:** antidepressants, antidepressant response, genetic pathway analysis, hippocampal neurogenesis, hippocampal volume, transcriptomics

## Abstract

Antidepressant‐induced hippocampal neurogenesis (AHN) is hypothesized to contribute to increases in hippocampal volume among major depressive disorder patients after long‐term treatment. Furthermore, rodent studies suggest AHN may be the cellular mechanism mediating the therapeutic benefits of antidepressants. Here, we perform the first investigation of genome‐wide expression changes associated with AHN in human cells. We identify gene expression networks significantly activated during AHN, and we perform gene set analyses to probe the molecular relationship between AHN, hippocampal volume, and antidepressant response. The latter were achieved using genome‐wide association summary data collected from 30,717 individuals as part of the ENIGMA Consortium (genetic predictors of hippocampal volume dataset), and data collected from 1,222 major depressed patients as part of the NEWMEDS Project (genetic predictors of response to antidepressants dataset). Our results showed that the selective serotonin reuptake inhibitor, escitalopram evoked AHN in human cells; dose‐dependently increasing the differentiation of cells into neuroblasts, as well as increasing gliogenesis. Activated genome‐wide expression networks relate to axon and microtubule formation, and ribosomal biogenesis. Gene set analysis revealed that gene expression changes associated with AHN were nominally enriched for genes predictive of hippocampal volume, but not for genes predictive of therapeutic response.

## 1 INTRODUCTION

Neurogenesis is the process by which new neurons are formed from differentiating neural progenitor cells in the brain (Götz & Huttner, 2005). It is a process critical for neurodevelopment in utero, infancy and adolescence (Johnson, 2001). Unlike most brain regions, the subgranular zone of the dentate gyrus within the hippocampus has a pool of neural progenitor cells that continue to show neurogenic activity throughout adulthood, across a number of species, including humans (Eriksson et al., 1998). This gives rise to hippocampal neurons possessing uniquely plastic properties, including a reduced threshold for long term potentiation and the ability to form new synaptic connections within existing mature neuronal architecture (Eriksson et al., 1998). Notably, levels of neurogenesis are affected by environmental factors (Lledo, Alonso, & Grubb, 2006). In rodent studies, stress hormones, immuno‐inflammatory activation, diet, exercise and medications have been reported to impact upon the rate of hippocampal neurogenesis; with subsequent effects on learning, memory and the regulation of mood (Murphy, Dias, & Thuret, 2014; Musaelyan et al., 2014; Potts & Lim, 2012; Praag, Kempermann, & Gage, 1999; Praag et al., 2002; Schoenfeld & Gould, 2012; Strangl & Thuret, 2009).

Reductions in hippocampal volume are also found across a number of psychiatric disorders, most notably among major depressive disorder patients (Videbech & Ravnkilde, 2004). Studies have revealed that antidepressants increase hippocampal neurogenesis in non‐human animal models, and more recently in human hippocampal progenitor cells (Anacker et al., 2011; Berton & Nestler, 2006). Antidepressant‐induced hippocampal neurogenesis (AHN) has been suggested as one important cellular mechanism contributing to an increase in hippocampal volume after long‐term treatment (Malykhin, Carter, Seres, & Coupland, 2010). However, due to the inaccessible nature of hippocampal progenitor cells in patients, there has been no conclusive evidence directly supporting this relationship in humans. Furthermore, based on convincing non‐human animal data, AHN has been implicated as a cellular mechanism governing antidepressant action (Santarelli et al., 2003) but this, to our knowledge, has yet to be demonstrated in humans.

Using a multidisciplinary approach accessing human hippocampal stem cells and leveraging large cohort datasets, the current study attempted to extend our understanding in the field by addressing two aims. First, we aimed to characterize genome‐wide expression networks activated during early AHN in human cells treated with the selective serotonin reuptake inhibitor (SSRI), escitalopram. Secondly, based on our expression results we aimed to identify a gene set associated with AHN. We then tested whether this gene set showed associative enrichment within genome‐wide association studies relating to: (i) hippocampal volume, and (ii) antidepressant response. A significant enrichment would suggest that AHN was activating pathways relevant to each phenotype, and may represent the cellular mechanism contributing to each phenotype.

## 2 METHODS

### 2.1 The hippocampal progenitor cell line

The multipotent, human hippocampal progenitor cell line HPC0A07/03C (provided by ReNeuron, Surrey, UK) was used for all experiments, as described previously (Anacker et al., 2011). ReNeuron's HPC0A07/03C cells were obtained from a 12‐week‐old fetus and immortalized with c‐mycER technology. In the presence of growth factors (FGF2 and EGF) and 4‐OHT, progenitors cells will proliferate indefinitely. Removal of these growth factors induces differentiation of cells into hippocampal neurons, astrocytes, and oligodendrocytes, but not into other cell types outside of the brain. Cells were grown in Dulbecco's Modified Eagle's Media/F12 (DMEM:F12, Invitrogen, Paisley, UK) supplemented with 0.03% human albumin (Baxter Healthcare, Compton, UK), 100 μg/ml human apo‐transferrin (Sigma, St‐Louis, MO), 16.2 μg/ml human putrescine DiHCl (Sigma), 5 μg/ml human insulin (Sigma), 60 ng/ml progesterone (Sigma), 2 mM l‐glutamine (Sigma) and 40 ng/ml sodium selenite (Sigma). To maintain proliferation, 10 ng/ml human bFGF (Pepro Tech EC Ltd, London, UK), 20 ng/ml human EGF (Pepro Tech EC Ltd.) and 100 nM 4‐OHT (Sigma) were added in proliferating media and removed in differentiating media. The cell line underwent routine checking for mycoplasma contamination every 6 weeks. All cells were grown at 37°C, 5% CO_2_, and in a humidified atmosphere. Within these sets of experiments each “biological replicate” represents a subculture of cells obtained from a different passage.

### 2.2 Drug dose

The active metabolite of the SSRI escitalopram, (S)‐citalopram, has a therapeutic window of between 50 and 130 ng/ml (Kumar, Kung, & Shinozaki, 2014), which corresponds to doses of between 120 and 313 nM in vitro. Subsequently, cells were treated with a range of doses, incorporating this therapeutically‐relevant window. Drug doses were achieved by dissolving escitalopram oxalate (Sigma) in molecular grade ethanol (Sigma) to form a 10 mM stock solution. Drug doses (145 nM, 290 nM, and 1160 nM) were then formed by dilution of the stock with media; with the relative proportion of ethanol kept constant across all dose groups including a vehicle control (0 nM).

### 2.3 Culture protocol

Our cell culture protocol was adapted from one described previously, in which hippocampal neurogenesis was observed when cells were treated with an SSRI during both the proliferative growth stage and during the cell differentiation stage (Anacker et al., 2011). However, we shortened this protocol from a 10‐day drug treatment protocol to 9 days, in order to capture very early changes in neurogenesis.

Cells were seeded for 24 hr on laminin‐coated 96 well (immunocytochemistry experiments) or six well (gene expression experiments) plates (Nunclon, Roskilde, Denmark). After seeding, cells were treated with proliferating media containing varying doses of escitalopram oxalate for 48 hr. At the end of the 48‐hr drug treatment period, proliferating media was aspirated. Media was then replaced with differentiating media (lacking EGF, bFGF, 4‐OHT) twice over 1 hr to ensure thorough removal of growth factors. Subsequently, this media was replaced with differentiating media containing corresponding doses of escitalopram, as in the proliferation protocol. The cells were grown in differentiating media for 7 days during which they differentiated into doublecortin and Map‐2 positive neurons (See Supplementary Information). Subsequently, cells underwent fixing (96‐well plates) and RNA collection (6‐well plates). 96‐well plates were fixed in 4% paraformaldehyde for 20 min at room temperature, and then stored in phosphate buffered saline at 4°C for later immunocytochemistry experiments. For cells grown in 6‐well plates, media was aspirated and 1 ml of Trireagent (Sigma) was added for later RNA isolation.

### 2.4 Immunocytochemistry

For Immunocytochemistry experiments, three biological replicates and three technical replicates were utilized for each staining condition, *n* = 12 in total per staining condition; which is comparable to other studies identifying moderate to large effect sizes using this cell line (Anacker et al., 2011). The primary outcome measures of interest were changes to Doublecortin (Dcx) staining, that is, the differentiation of progenitor cells into to neuroblasts, and Map‐2, that is, a marker for more mature neurons. However, we also assessed other markers assaying proliferation, using BrdU and Ki67 staining; changes to gliogenesis using S100β staining; and cell death, using cleaved caspase‐3 (CC3) staining. For more details, see S1 and S2.

### 2.5 Transcriptomics

RNA from cell experiments was isolated using Trireagent (Sigma) following the standard protocol, with an additional ethanol precipitation step to increase RNA purity. For gene expression experiments we utilized six biological replicates at four drug doses (*n* = 24 total). RNA samples were processed on Illumina Human HT‐12 v4 Expression BeadChip (Illumina Inc., San Diego, CA) according to manufacturer's protocol. For more details, see S3.

### 2.6 Statistical analysis

#### 2.6.1 Immunocytochemistry experiments

For each biological replicate, percentage change in marker immunofluorescence from our drug treated cells relative to controls was calculated for each technical triplicate and the mean was taken. Linear regressions were then used to assess the relationship between percentage change in marker immunofluorescence and: (i) drug/no drug (dose groups collapsed), and (ii) drug dose, whilst co‐varying for staining batch. Within each of the four staining groups targeting different biological questions (differentiation: Dcx, Map‐2; proliferation: BrdU, Ki67; gliogenesis: S100β; cell death: CC3) we applied a Bonferroni correction for the number of tests. We consider corrected *P* values of *P* ≤ 0.05 as significant. See Supplementary Materials for more information.

#### 2.6.2 Transcriptomic experiments

Probes were normalized using a systematic approach incorporating the Lumi package (Du, Kibbe, & Lin, 2008) and analyzed using R (www.r-project.org). Probes were filtered to remove lowly detected probes and to investigate only the 7,500 most variable probes, see Supplementary Materials. For transcriptomic studies we focused on the dose‐dependent effects of drugs on gene expression only. A linear regression was performed, where probe expression was selected as the dependent variable, dose as the independent variable, and batch and biological replicate included as factors. A total sample number of 24 provided 80% power to detect a 1.5 fold change in expression as part of a two‐tailed *t*‐test at a level of significance corrected for multiple comparisons (*p*_corr_ = 0.05/7500 = *P* ≤ 6.6E‐6), with an estimated standard deviation of 0.5. For more details, see S4.

#### 2.6.3 Gene network analysis

To understand which biological mechanisms may be affected by drug dose, in a hypothesis‐free manner, we used Webgestalt (bioinfo.vanderbilt.edu/webgestalt/). All probes nominally predicted by drug dose (*P* ≤ 0.05) were tested for Gene Ontology (version 1.2) term enrichment with Biological Processes (GOTERM_BP_FAT), Cellular Components (GOTERM_CC_FAT), Molecular Function (GOTERM_MF_FAT) and KEGG pathways. We utilized all probes surviving background correction as our comparison group or “background list.” We further entered genes within enriched pathways of interest into Gene Mania (http://www.genemania.org), in order to visualize gene expression networks.

#### 2.6.4 Genetic pathway analysis

We identified a gene set by selecting all genes showing nominally significant expression changes (*P* ≤ 0.05) in our data set, and we tested if our gene set was enriched for associative signal with two phenotypes: (i) hippocampal volume in adults; (ii) antidepressant response. We achieved this using the pathway analysis tool, MAGMA (Leeuw, Mooij, Heskes, & Posthuma, 2015), with a 35 kb 5′ and 10 kb 3′ window around genes and the results from two large genome‐wide association studies (GWAS). There were genome‐wide summary statistic data from the ENIGMA Consortium, which performed a meta‐analysis identifying the genetic predictors of hippocampal volume in a total of 30,717 subjects (http://enigma.ini.usc.edu) (Hibar et al., 2015) and results from the NEWMEDS project, which has investigated genetic predictors of antidepressant response (Tansey et al., 2012). Within NEWMEDS we utilize the GWAS results specifically relating to SSRIs, which is derived from a total of 1,222 major depressive disorder patients (Tansey et al., 2012). Antidepressant response was defined as an adjusted continuous measure capturing change in depression severity after taking an SSRI for a number of weeks (see Tansey et al. (2012), for more details).

MAGMA tests for gene set enrichment by first generating a gene‐wide statistic from the GWAS results files, adjusting for gene size, SNP density and linkage disequilibrium effects (Leeuw et al., 2015). MAGMA then performs a competitive test of gene set association to investigate if associative enrichment within a user‐defined gene set, outperforms other gene sets from across the genome of similar size (Leeuw et al., 2015). Due to the stringent nature of the competitive test within MAGMA, the fact that the two phenotypes under investigation relate to two separate biological questions, and the fact the genome‐wide datasets consist of vastly different sample sizes, we considered a *P*‐value threshold of *P* ≤ 0.05 as significant.

## 3 RESULTS

### 3.1 Immunocytochemistry

#### 3.1.1 Effects of escitalopram on neuronal differentiation

**Figure 1:**
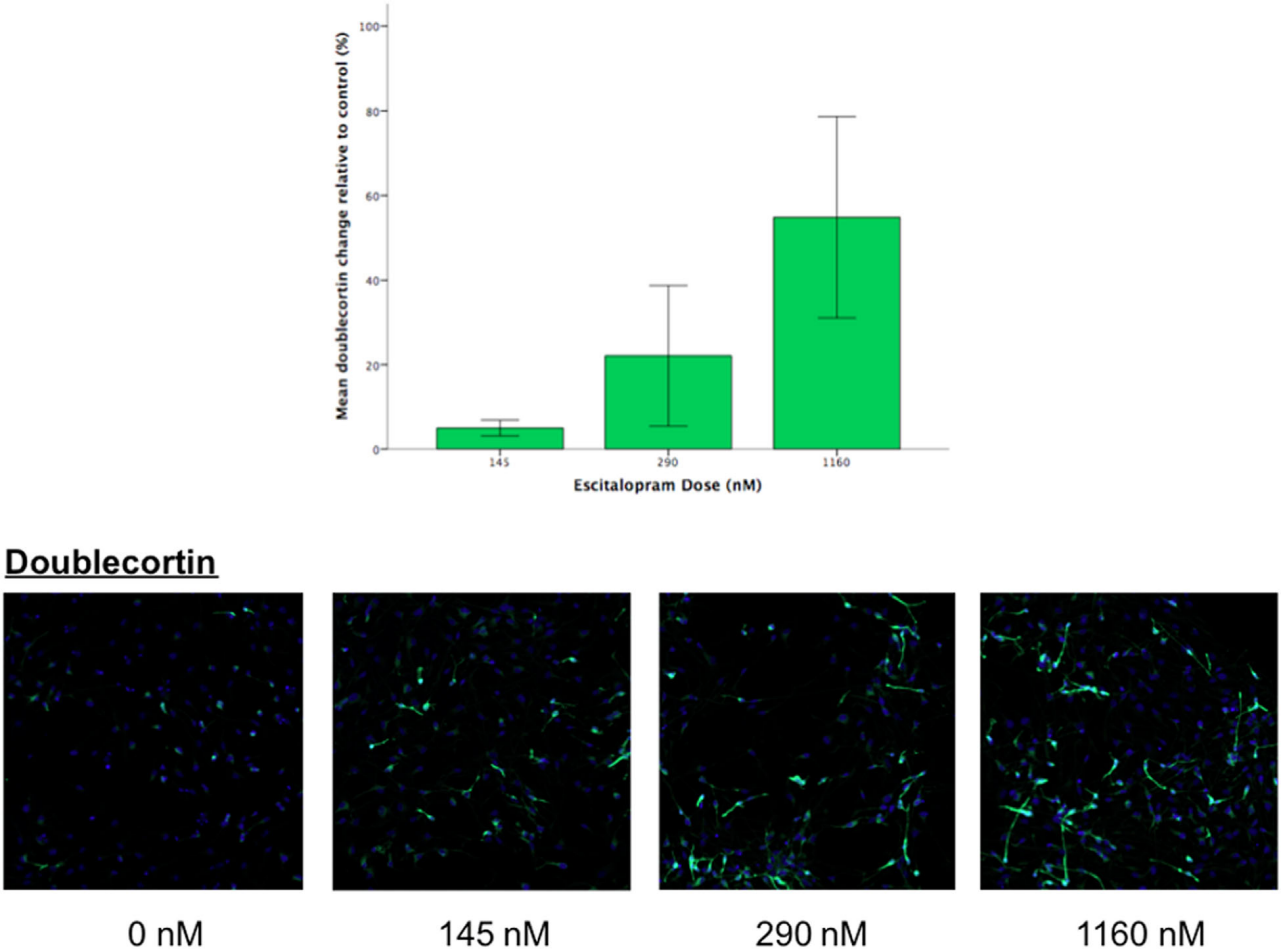
Top: Bar chart shows that the mean percentage of cells expressing doublecortin dose‐dependently increases relative to control conditions in differentiating cells treated with escitalopram, based on linear regressions, covarying for staining batch (*P* ≤ 0.01, *n* = 12). Error bars represent the standard error of the mean. Bottom: Corresponding images of cell nuclei (blue) and marker immunofluorescence (green) in differentiating cells treated with escitalopram at each drug dose. [Color figure can be viewed at wileyonlinelibrary.com]

Doublecortin staining was used to measure early changes in the differentiation of progenitor cells into immature neurons (neuroblasts) and Map‐2 was used to measure changes in the differentiation of more mature neurons. Results revealed a dose‐dependent increase in the differentiation of cells into neuroblasts as characterized by the marker doublecortin (*F* = 15.014, d.f. = 1, *P* = 0.006), see Figure 1. This effect remained significant after multiple testing correction (4 tests; corrected *P* = 0.024). There were no other significant changes (*P *> 0.05).

#### 3.1.2 Effects of escitalopram on other cell markers

BrdU and Ki67 were used to assess changes in proliferation. Our staining also revealed significant increases in BrdU staining in cells (independent of dose) treated with escitalopram (*F* = 9.358, d.f. = 1, *P* = 0.018), but this effect was not significant after multiple testing correction (4 tests; corrected *P* > 0.05).

S100β was used to assess gliogenesis. We found increases in S100β in cells treated with escitalopram, independent of dose (*F* = 28.813, d.f. = 1, *P* = 0.001), which remained significant after multiple testing correction (2 tests; corrected *P* = 0.002).

CC3 staining confirmed that the drug/drug doses were not inducing cell death (uncorrected *P* > 0.05). See Supplementary Information for more details.

### 3.2 Transcriptomics

Our results revealed one genome‐wide significant expression change in the *Nucleolar protein 6* (*NOL6*) gene (Probe: ILMN_7349, *t* = 6.485, S.E. = 3.376e‐05, *P* = 5.600e‐06), which showed a dose‐dependent increase in expression, see S8.

### 3.3 Gene ontology and KEGG pathway analysis

**Figure 2:**
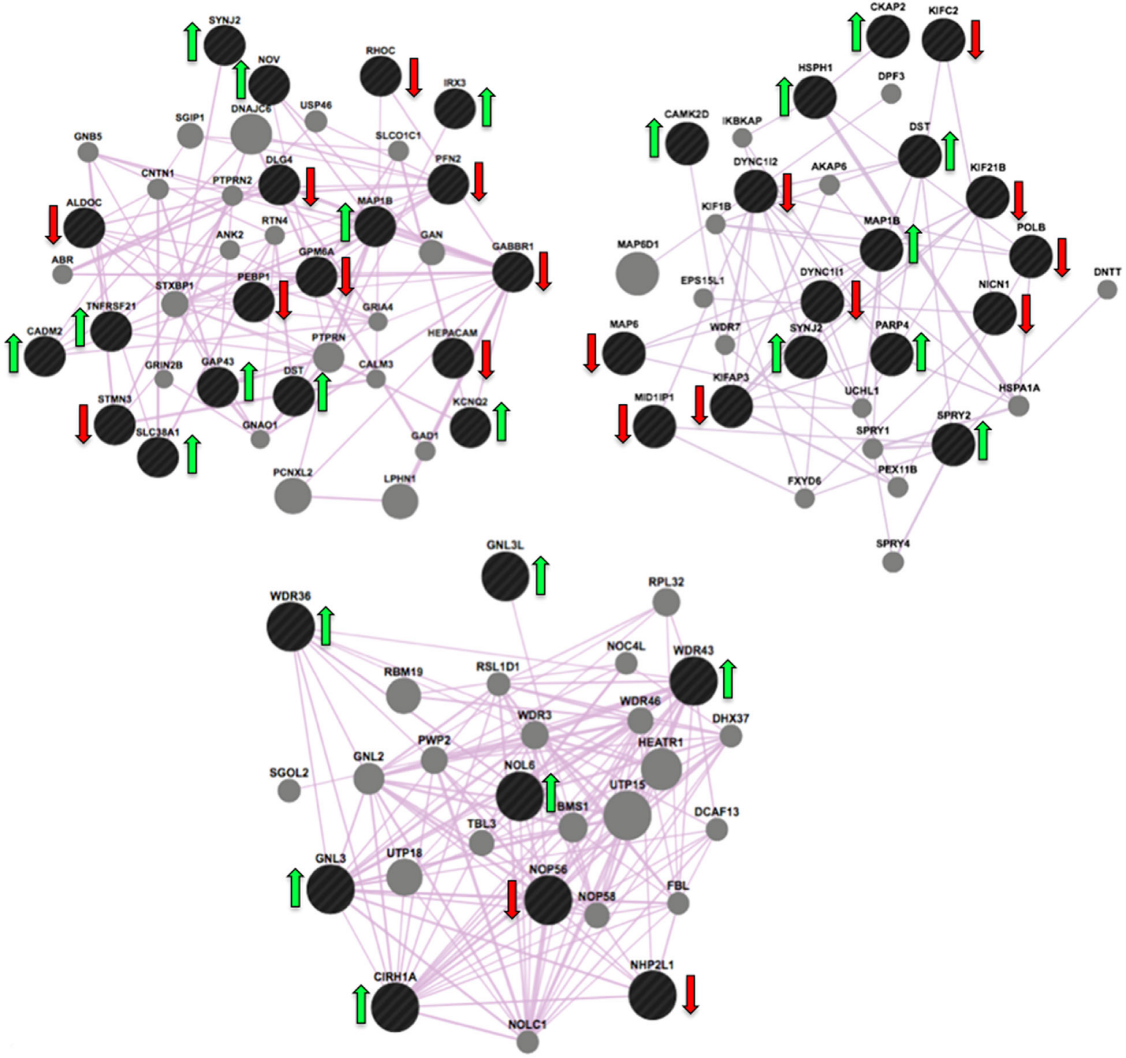
Top Left: Gene expression networks activated whilst differentiating cells are treated with varying doses of escitalopram. These figures show transcripts from our experimental data (black) and predicted transcripts (grey) involved in three pathways which show enrichment in our data: (i) Axon (GO:0030424), top left; (ii) Microtubule (GO:0005874), top right; and (iii) Ribosomal Biogenesis (KEGG Pathway), bottom. Genes with an adjacent green arrow represent those showing an increase in expression in response to escitalopram dose, genes marked with a red arrow represent those showing a decrease in expression. [Color figure can be viewed at wileyonlinelibrary.com]

545 probes were nominally associated with drug dose (*P* < 0.05) and were input into Webgestalt. There were a total of 13 Bonferonni corrected GO terms associated with drug dose, see Table 1. Results from KEGG pathway analysis identified an enrichment in the “Ribosome biogenesis in eukaryotes” pathway (Enrichment Ratio = 5.28; Corrected *P* = 0.0045; Figure 2).

**Table 1:** Gene Ontology (GO) terms significantly enriched in gene expression data generated while differentiating hippocampal progenitor cells are treated with escitalopram, sorted by enrichment ratio

Database	GO name	GO ID	Enrichment ratio	Corrected *P*‐value
Cellular component	Axon	GO:0030424	2.94	0.0028
Cellular component	Microtubule	GO:0005874	2.57	0.0164
Molecular function	RNA binding	GO:0003723	1.88	0.0382
Cellular component	Mitochondrion	GO:0005739	1.66	0.0153
Cellular component	Cytoplasm	GO:0005737	1.23	2.39E‐05
Cellular component	Cytoplasmic part	GO:0044444	1.22	0.0328
Molecular function	Protein binding	GO:0005515	1.19	0.0382
Cellular component	Intracellular membrane‐bounded organelle	GO:0043231	1.16	0.0328
Cellular component	Intracellular	GO:0044424	1.15	6.61E‐05
Cellular component	Intracellular organelle	GO:0043229	1.15	0.008
Cellular component	Organelle	GO:0043226	1.15	0.0094
Cellular component	Intracellular	GO:0005622	1.14	6.15E‐05
Molecular function	Binding	GO:0005488	1.09	0.0382

Data were generated by inputting 545 probes nominally associated with drug dose (*P *< 0.05) into Webgestalt.

### 3.4 AHN gene set and its relationship to hippocampal volume and antidepressant response

545 probes were nominally associated with drug dose (*P* < 0.05) and MAGMA tested for enrichment of this gene set within GWAS datasets. In our first analysis, we found a nominally significant enrichment of our AHN gene set in GWAS results relating to hippocampal volume (competitive *P* = 0.031). However, the AHN gene set did not show a significant enrichment within the antidepressant response GWAS data (competitive *P* = 0.464).

## 4 DISCUSSION

Our results show that escitalopram dose‐dependently accelerated the differentiation of human hippocampal progenitor cells into neuroblasts, Figure 1, whilst also increasing gliogenesis. Our findings in human cells are supported by rodent studies, which have shown that escitalopram treatment correlates with increases in hippocampal cytogenesis (Jayatissa, Bisgaard, Tingström, Papp, & Wiborg, 2006). Our immunocytochemistry data are also complemented by our corresponding gene expression data, which revealed “Axon” and “Microtubule” as two significant GO terms with the highest enrichment ratios, Table 1. Within these networks, the transcripts of gamma‐aminobutyric acid B receptor, 1 (*GABBR1*) and Microtubule‐associated protein 1B (*MAP1B*) act as hub genes, suggesting they may represent important drug targets, Figure 2. Indeed, the reduction in *GABBR1* expression found in our study is corroborated by other reports showing that a reduction in GABA receptor binding stimulates neurogenesis (Giachino et al., 2014). In contrast, *MAP1B* shows an increase in expression in response to escitalopram dose. MAP1B is a protein involved in microtubule assembly; a process essential for mediating neurogenesis (Beermann & Shea, 1994).

The KEGG pathway analysis revealed that “Ribosome biogenesis in eukaryotes” was also an enriched pathway, and features Nucleolar protein 6 (*NOL6*) as a hub gene; the transcript most significantly affected by escitalopram dose, showing a dose‐dependent increase in expression, Figure 2. Ribosomal biogenesis involves the synthesis of the machinery involved in polypeptide formation, and subsequently determines the translational capacity of a cell (Bernstein, Gallagher, Mitchell, Granneman, & Baserga, 2004). Our findings corroborate previous studies implicating increases in ribosomal biogenesis in cells undergoing increases in proliferation, neurite formation and synaptogenesis (Hsieh, 2012).

In our hypothesis‐driven genetic pathway analysis, we found that our AHN gene set was nominally enriched for genes predicting hippocampal volume in adults. This suggest that increases in hippocampal volume observed after long‐term antidepressant treatment are likely mediated through the activation of neurogenic pathways, as opposed to solely a reduction in neuronal cell death. However, our AHN gene set was not enriched for genes predictive of antidepressant response, suggesting that in contrast to non‐human animal findings (Santarelli et al., 2003), AHN may not be the cellular mechanism governing therapeutic response to SSRIs in humans. There are, however, a number of alternative interpretations to this finding. For example, rather than a direct causal link between AHN and antidepressant response, AHN might simply facilitate therapeutic response through increasing “plasticity” allowing social support networks and positive, enriching environmental factors to have an enduring effect on the brain and mood of patients (Spalding et al., 2013). Indeed, non‐human studies have shown that enriched environments (e.g., increases in running) can moderate hippocampal neurogenesis and evoke antidepressant effects (Spalding et al., 2013; van Praag, Kempermann, & Gage, 1999). Consequently, the heterogeneous environments experienced by patients outside of the clinic may moderate the impact of AHN on therapeutic response, preventing us from identifying a direct genetic relationship.

Although our findings are promising, this study has several limitations. First of all, an in vitro model cannot fully account for the milieu of environmental factors present in human major depressive disorder patients (hormones, diet, levels of physical activity), which can moderate hippocampal neurogenesis. Secondly, the human hippocampal progenitor cell line used is fetal in origin and results obtained from this study may not reflect how adult cells respond to antidepressants. Thirdly, the differentiation protocol utilized here was relatively short, which allowed us to characterize very early AHN, as marked by changes to doublecortin but not Map‐2. Future studies could also investigate gene expression changes caused by escitalopram during neuronal maturation using a longer protocol. Fourth, our ability to detect enrichment of our AHN gene set for genes relating to hippocampal volume (ENIGMA study) but not antidepressant response (NEWMEDS study) may relate to power. For instance, the ENIGMA sample utilized an objective phenotype (hippocampal volume) and a large sample size (*n* = 30,717), giving it excellent power to detect genetic predictors. In contrast, the NEWMEDS sample suffers from the fact it measures a more subjective and heterogeneous phenotype (antidepressant response) in a sample size (*n* = 1,222) over twenty times smaller than the ENIGMA cohort. Consequently, in order to draw firmer conclusions regarding the relationship between AHN and antidepressant response, a larger patient sample size is needed in future studies.

Despite these limitations, this study is the first to: (i) find that escitalopram increases neurogenesis in human hippocampal progenitor cells; (ii) identify gene expression networks activated during AHN; (iii) use genetic pathway analysis to reveal that AHN gene expression changes are nominally enriched for genes predictive of adult hippocampal volume, but not genes for antidepressant response.

## Supporting information

Additional supporting information may be found in the online version of this article at the publisher's web‐site.

Supporting Data S1.
