# Supplementary material for: The genome‐wide expression effects of escitalopram and its relationship to neurogenesis, hippocampal volume, and antidepressant response
Source: Am J Med Genet B Neuropsychiatr Genet. 2017 Apr 10;174(4):427–34. doi: 10.1002/ajmg.b.32532 (PMC5485083; doi:10.1002/ajmg.b.32532)
Supplement: Supplementary file 1 — Supporting Data S1. [file AJMG-174-427-s001.docx]

**Supplementary Materials**

**S1 BrdU Incorporation and Immunohistochemistry**

Once fixed, wells containing BrdU were treated with 50 μL of 2 N HCL for 25 minutes to allow antibodies access to the nucleus. Wells were then neutralised with 100 μL of 0.1 M borate buffer for 10 minutes and washed twice with PBS. For all wells, 50 μL of blocking solution (0.3% Triton X-100, 5% normal donkey serum (Alpha Diagnostics, San Antonio, TX, USA) in PBS) was added for 1 hour and left at room temperature. Primary antibodies were subsequently diluted in blocking solution and added in 30 μL per well and left overnight at 4°C. Proliferation was assessed both in proliferating and differentiating cells using antibodies targeting bromodeoxyuridine (BrdU) (Rat, anti-BrdU, 1:500, Serotec, Oxford, UK, Catalogue no. OBT0030CX) and Ki67 (Rabbit; anti-Ki67; 1:500; Abcam, Cambridge, UK, Catalogue no. ab15580), as well as Cleaved Caspase-3 antibodies which were used as a marker of cell death (Rabbit; anti-CC3; 1:500; Cell Signaling Technology, Danvers, MA, USA, Catalogue no. ♯9664). Differentiation was only assessed in differentiating cells only using doublecortin (Dcx), which is present in immature neuroblasts (Rabbit; anti-Dcx; 1:500; Abcam, Catalogue no. ab19723), and microtubulin-associated protein-2 (MAP2) which is present in more mature neurons (Mouse; anti-Map2; 1:500; Abcam, Catalogue no. ab11267). Gliogenesis was characterised using S100 calcium-binding protein β (S100β) in differentiating cells (Rabbit; anti-S100β; 1:500; Dako, Glostrup, Denmark, Cataloge no. 20311).

The following day, primary antibody solutions were removed and washed twice with PBS. Wells were then incubated for 30 minutes at room temperature in blocking solution. Following this, secondary antibodies diluted in 30 μL of blocking solution were added and allowed to incubate at room temperature for 2 hours. Secondary antibodies used were all manufactured by Invitrogen and were diluted in 1:500. Secondary antibodies include: Alexa 488 goat anti-rat, Alexa 555 goat anti-rabbit, Alexa 488 anti-rabbit and Alexa 555 goat anti-mouse. Wells were then washed twice with PBS and 50 μL 4',6-diamidino-2-phenylindole (DAPI; 1:2000; Sigma) was added per well (for nuclear staining). Wells were then washed three times with PBS and stored in a final third wash at 4°C in preparation for immunofluorescence detection. The CellInsight NXT High Content Screening (HCS) Platform (ThermoScientific, Wilmington, DE, USA) was used for immunofluorescence detection.

**S2 Cell Insight: Characterisation of Immunofluorescence**

We used two BioApplication software packages (ThermoScientific, UK): (i) Target activation enables assessment of nuclear stainings e.g. BrdU and Ki67 and (ii) Cell Health Profiling to quantify CC3, S100β, Dcx, and Map2. Target activation facilitates the assessment of co-labelling within the region of the nucleus. Cell health profiling involves construction of a concentric circle around the nucleus, to create a map or region of interest. Staining confined within the region between the outer circle (edge of cell body) and inner circle (outside of the nucleus) is deemed positive.

Based on values from the negative staining controls (for each secondary antibody) and positive staining, distinct and robust thresholds are set for average intensity to delineate positive populations for each marker across all experimental conditions. The threshold set for positive staining must be higher than the highest value observed in the negative staining control. A defined and stringent threshold identifies positive staining in an automated and unbiased way. For each marker we obtained data collected from 15 non-overlapping fields per well.

**S3 RNA Quality and Microarray Array Preparation**

All RNA samples had 260/280 ratios of above 1.75, as assessed using the Nanodrop ND1000 (Thermoscientific). RNA integrity numbers (RINs) were assessed using the Agilent Bioanalyzer (Agilent Technologies, Berkshire, UK) and all samples had RINs of greater than 9. The current study utilised six biological replicates at four drug doses (n=24 total). RNA samples were processed on Illumina Human HT-12 v4 Expression BeadChip (Illumina Inc., San Diego, CA) according to manufacturer’s protocol. Complementary DNA was synthesized using 300ng of total RNA followed by amplification and biotinylation of complementary RNA and hybridization according to the protocol supplied with the Illumina Total-Prep RNA Amplification Kit (Life Technologies, UK).

**S4 Microarray Preprocessing**

Initial quality control assessment was performed in Genome Studio, where outliers were identified using a scatterplot of average signal intensities. One sample was revealed as an outlier, and this was confirmed using hierarchical clustering. This sample was subsequently removed from downstream analysis. The Lumi (Bioconductor) package in R (<http://www.R-project.org>) was used for quality control, quantile-normalization, log-transformation and gene annotation. Genes were then filtered based on detection values generated by Genome Studio. Expression probes had to reach the detection p-value threshold <0.01 in at least one sample, and if not, they were excluded. We further filtered out any probes showing little to no variation across each condition in order to reduce the burden of multiple testing correction, utilizing only the 7,500 most variable probes per group.

**S5 Cell Images**


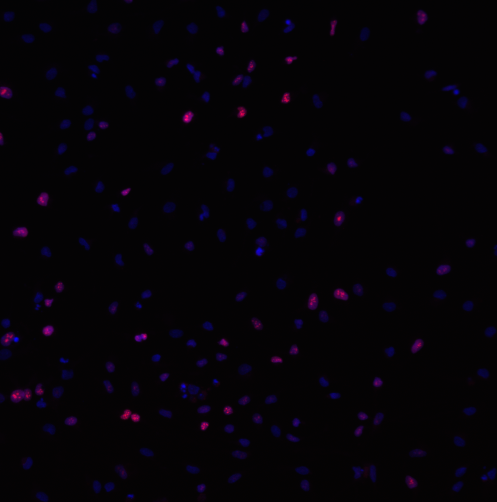
**Map2 Ki67**


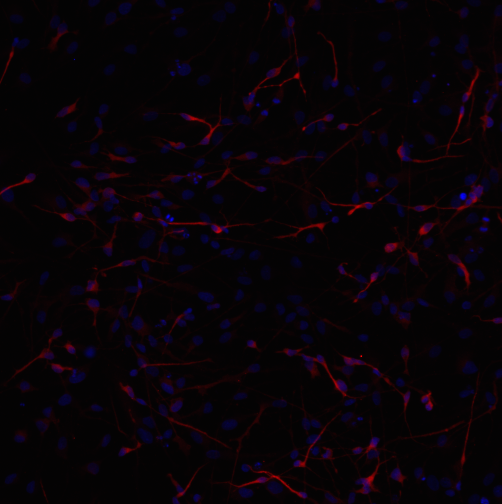


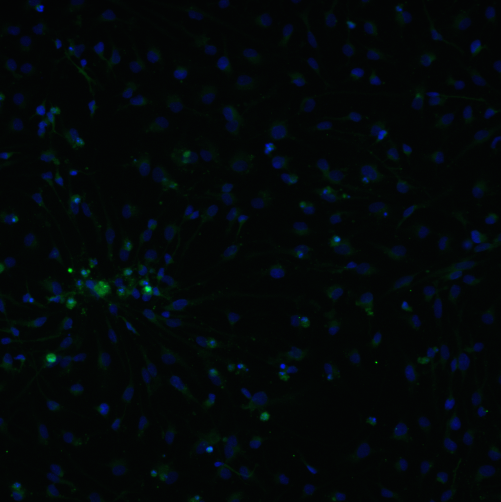


**CC3**

**BrdU**


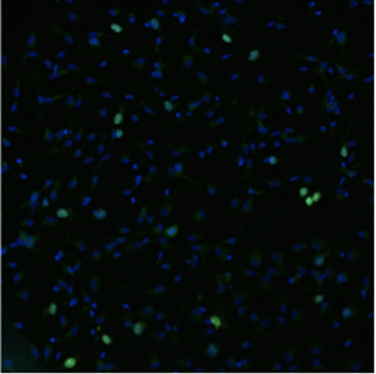


**S100β**


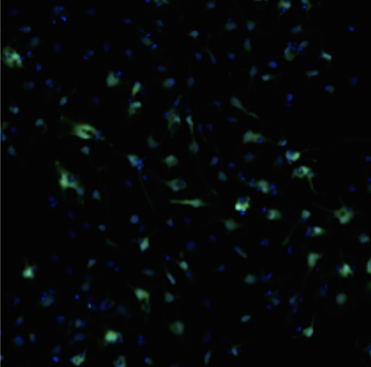


S5: Representative images of other cell markers assayed (not shown in main text). Nuclei are stained in blue, with markers indicated by the title.

**S6 Immunohistochemistry data analysis**

Percentage difference in marker immunofluorescence from our drug treated cells relative to controls was calculated for each technical triplicate. Linear regressions were used to assess the relationship between drug/no drug (dichotomous), and drug dose on marker immunofluorescence.

The mean of the three technical replicates was then calculated, however if there was a standard deviation of greater than 2, we utilised the two technical replicates generating the lowest standard deviation. The average percentage change per marker calculated for each of the three biological replicates at each dose were then utilised as the independent variable in linear regressions. Normality was confirmed based on skewness and kurtosis values residing within values of +/- 1.5.

First, to assess whether drug (independent of dose) had an effect on marker immunofluorescence, we performed a linear regression with percentage change in immunofluorescence chosen as the dependent variable, drug/no drug selected as the independent variable, with dose and staining batch included as covariates. Secondly, to assess whether drug dose had an effect on marker immunofluorescence, we performed a linear regression with percentage change in immunofluorescence chosen as the dependent variable, drug dose selected as the independent variable, with staining batch included as a covariate. P-values of P ≤ 0.05 were considered significant.

**S7 Full immunohistochemistry results**

**S8 Plots of gene expression data**


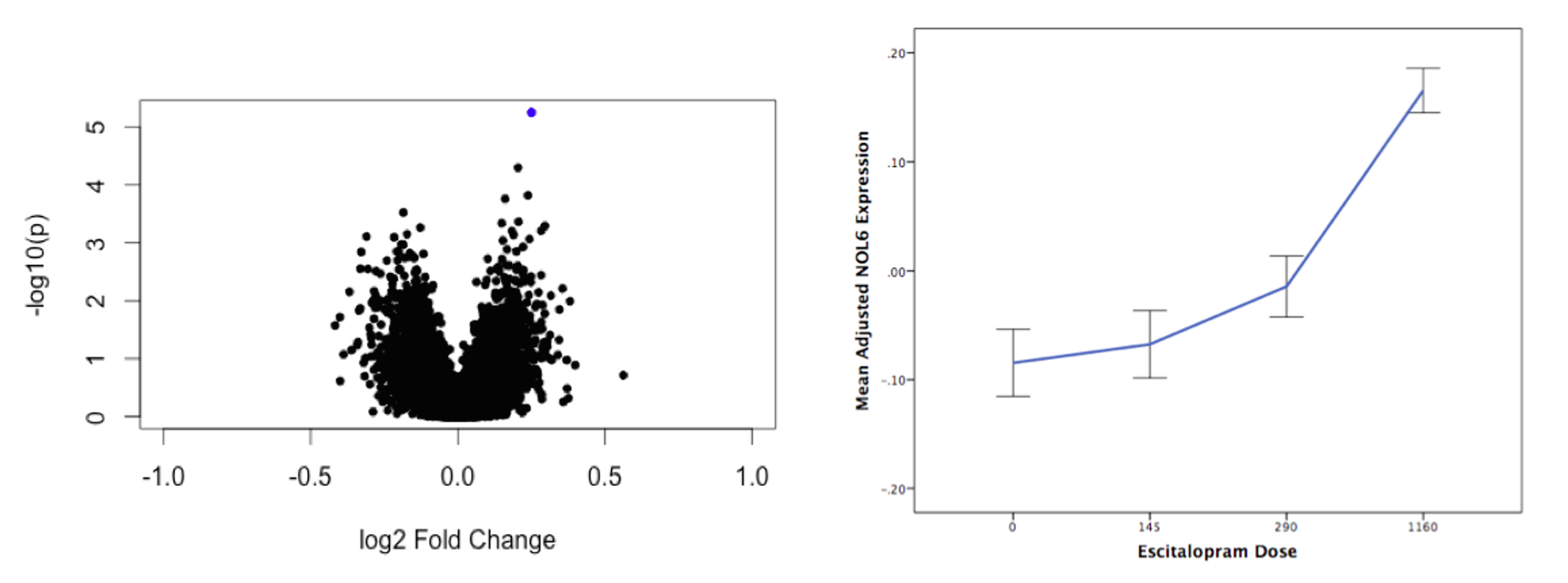


Left: A volcano plot displaying log2 fold changes (Mean Expression high dose – Mean expression control dose) and –log10 p-values generated from linear regressions using data generated from differentiating cells treated with varying doses of escitalopram. Data points represent each of the 7,500 most variable probes under investigation. The blue data point represents a probe assaying *NOL6*. Right: A line graph showing the expression of *NOL6* (y-axis) increases with escitalopram dose (x-axis) in differentiating cells after adjusting for the effects of biological replicate and array batch [P ≤ 6.6E-6].
